# A simple viability analysis for unicellular cyanobacteria using a new autofluorescence assay, automated microscopy, and ImageJ

**DOI:** 10.1186/1472-6750-11-118

**Published:** 2011-11-30

**Authors:** Katja Schulze, Diana A López, Ulrich M Tillich, Marcus Frohme

**Affiliations:** 1 Molecular Biology and Functional Genomics, TH Wildau, Bahnhofstr. 1, 15745 Wildau, Germany; 2 Bioinformatics, University of Wuerzburg, Biocenter, Am Hubland, 97074 Wuerzburg, Germany

## Abstract

**Background:**

Currently established methods to identify viable and non-viable cells of cyanobacteria are either time-consuming (eg. plating) or preparation-intensive (eg. fluorescent staining). In this paper we present a new and fast viability assay for unicellular cyanobacteria, which uses red chlorophyll fluorescence and an unspecific green autofluorescence for the differentiation of viable and non-viable cells without the need of sample preparation.

**Results:**

The viability assay for unicellular cyanobacteria using red and green autofluorescence was established and validated for the model organism *Synechocystis *sp. PCC 6803. Both autofluorescence signals could be observed simultaneously allowing a direct classification of viable and non-viable cells. The results were confirmed by plating/colony count, absorption spectra and chlorophyll measurements. The use of an automated fluorescence microscope and a novel ImageJ based image analysis plugin allow a semi-automated analysis.

**Conclusions:**

The new method simplifies the process of viability analysis and allows a quick and accurate analysis. Furthermore results indicate that a combination of the new assay with absorption spectra or chlorophyll concentration measurements allows the estimation of the vitality of cells.

## Background

The last couple of years have shown an increased research interest in microalgae and cyanobacteria, especially in the field of biofuels. Therefore simple and fast culture monitoring methods to determine the viability of the cells are needed.

As a simple method for the monitoring of fitness, absorption spectra give an overview of the amount of pigments and their distribution in the culture. Also the chlorophyll concentration (and the chlorophyll-concentration/OD_750_) can be used to analyze the culture condition [1]. Nevertheless, both methods only allow the quantification of growth and viability on the culture level, giving an average over all cells, but not a quantification of the viable cells. A differentiation of viable and non-viable cells on a cell by cell level allows for a causal evaluation of cell viability in regard to changing conditions, such as stress, cell aging or respiration rate. The standard method for the determining of cell viability is plating of the culture and subsequent counting of the colonies. This method however, requires plate preparation and at least one week until colonies can be identified. An alternative method for the determination of cell viability is fluorescent staining. In a dual-fluorescence assay the autofluorescence of chlorophyll is used to identify viable cells, and the fluorescent dye SYTOX Green is used to identify non-viable cells [2]. Although staining is faster than plating, it is vastly more expensive, and there is still need for culture preparation. For an automation of fluorescent staining, a flow cytometric approach for *Microcystis *has been previously reported [3]. The automation of the viability analysis via flow cytometry has the advantage of being less time consuming and more reliable. Nevertheless staining of the sample is still required. In contrast, the method presented in this paper could be adapted for use with flow cytometry and would allow the differentiation of viable cells without any sample preparation.

The new method is a fast and simple alternative using red chlorophyll fluorescence and an unspecific green autofluorescence for the determination of cell viability. Red autofluorescence of chlorophyll is only present in viable cells. For higher plants and filamentous cyanobacteria it is known, that there is a steady state between the building and degradation of chlorophyll. Senescence or dying results in a switch from equilibrium (with high turnover) to a massive degradation of chlorophyll [4,5]. Therefore the red autofluorescence fades and a green unspecific fluorescence, which can be observed at the same excitation wavelength but is superimposed by the red chlorophyll fluorescence in viable cells, becomes visible. This green fluorescence has mostly been described in higher plants and is caused by a variety of different molecules like flavonoids, flavins (e.g. FAD), cinnamic acids (e.g. ferulic acid), betaxanthine and pyridine nucleotides [e.g. NAD(P)H]. For algae this phenomenon has barely been analyzed and the molecules responsible for the green autofluorescence are mostly unidentified [6,7].

Nevertheless this effect allows one to distinguish between viable and non-viable cells (For this method non-viable cells are defined as cells that still show an intact cell shape but have lost the ability for growth and/or division.) at the same excitation frequency. By the use of a long pass filter for the emission wavelength both fluorescent signals can be observed simultaneously. A direct determination of the number of viable and non-viable cells is now possible without any sample preparation.

By the use of an automated fluorescence microscope and subsequent analysis of the generated images, an automated procedure for the determination of cell viability was created. For the image analysis, a plugin was written for ImageJ, an Open Source project which allows easy expansion [8,9]. The plugin enables the determination of the total cell concentration and the number of viable and non-viable cells by counting fluorescent cells and classing them by color.

We tested the red-green fluorescence assay for *Synechocystis *sp. PCC 6803, as it is a model for cyanobacteria and photoautotroph organisms in general [10]. The results for the automated viability analysis via ImageJ were validated with values obtained by plating, chlorophyll-measurement, and the analysis of the absorption spectra. Furthermore a temperature stressed *Synechocystis *culture was analyzed with the fluorescence assay and absorption spectroscopy. Results were compared with those of the viable culture, to test the applicability for a vitality analysis. Additionally the automated determination of the cell concentration by the plugin was investigated and compared to the optical density at 750 nm, which is often used to determine cell concentration for unicellular cyanobacteria.

## Results

Samples with 5*10^7 ^cells per ml, but different ratios of viable to non-viable cells were created. The percentage of viable cells and the total cell count per ml was then determined with the presented fluorescence method. Additionally the viable cell count via plating, chlorophyll content, the absorption spectra, and OD_750 _were determined for every sample to compare them to the results obtained with the new method.

### Fluorescence microscopy for viability analysis

**Figure 1:**
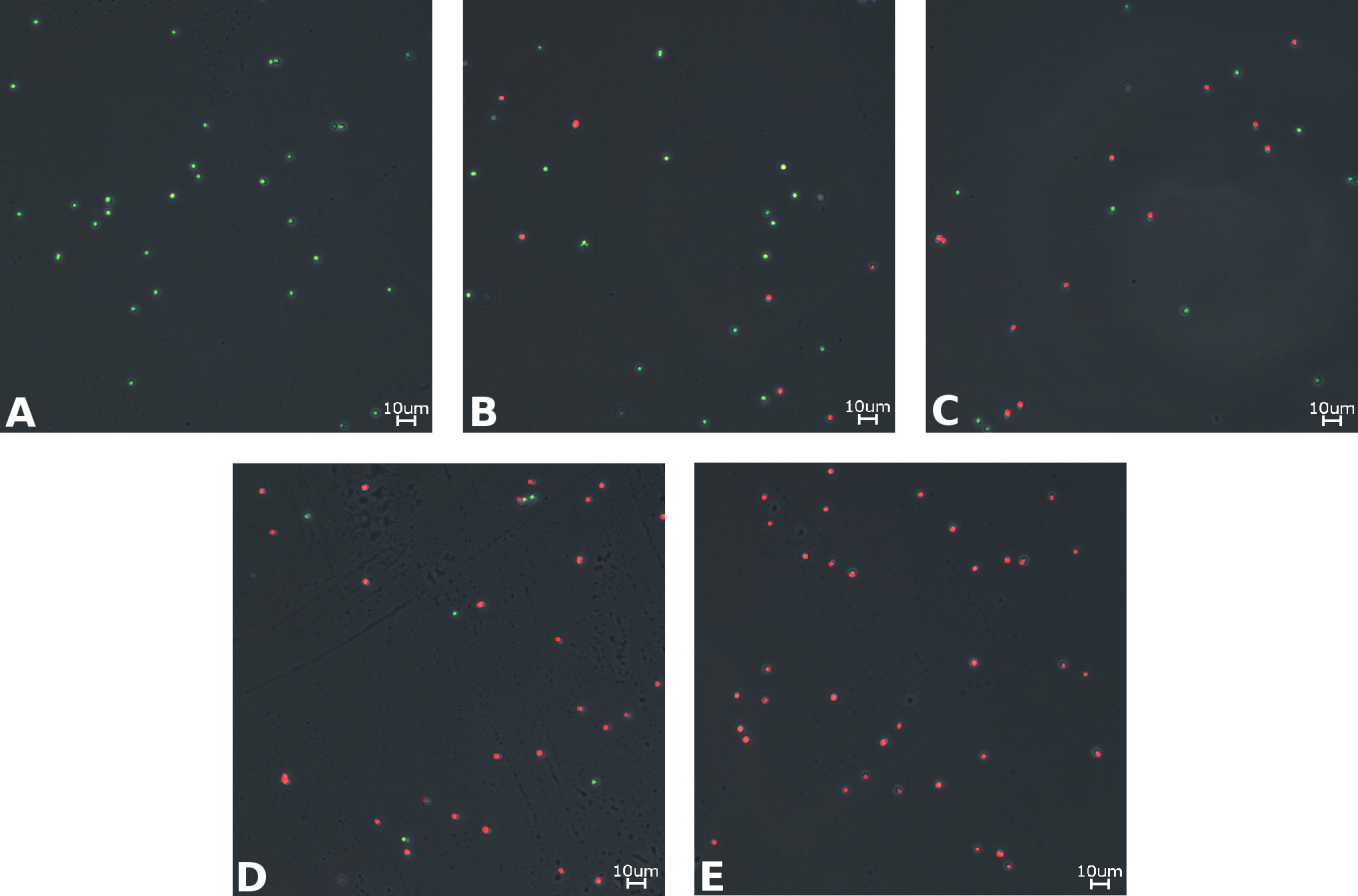
**Microscopic images of the *Synechocystis *samples**. Details of sample images for the examined *Synechocystis *samples with a different number of viable cells (A: 100%, B: 75%, C: 50%, D: 25%, E: 0%). All images show an overlay of microscopic fluorescent and phase contrast pictures. Red fluorescence is caused by chlorophyll and represents viable cells. Non-viable cells show a green unspecific autofluorescence.

**Figure 2:**
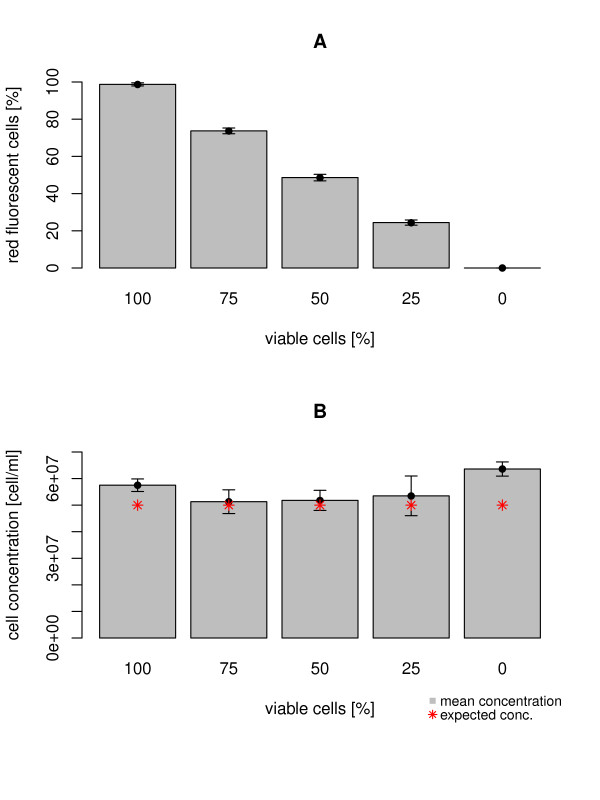
**Results for the cell-viability fluorescence assay**. The graph shows the determined number (A) and concentration (B) of red fluorescent cells for different samples containing a decreasing number of viable cells. Samples were analyzed three times using automated fluorescent microscopy and our plugin for ImageJ.

By using the autofluorescence of chlorophyll and an unspecific green autofluorescence of phototroph organisms, the proposed method allows a very easy determination of the number of viable cells of a culture. In all pictures the red fluorescent signal of the chlorophyll from viable cells showed a stronger intensity than the green signal from non-viable cells. Nevertheless a good detection of all signals was possible. The overlay of the fluorescent- and the phase contrast image confirms that the red (viable) and green (non-viable) fluorescent signal originate exclusively from *Synechocystis *cells (Figure 1). Additionally one can clearly observe a decrease of the number of red fluorescent cells and an increase of the number of green fluorescent cells for a decreased number of viable cells in the different samples. For all samples fluorescent images of a defined volume were taken in a Helber counting chamber in an automated procedure. With the subsequent analysis via ImageJ, the expected numbers of red (viable) and green (non-viable) cells could be determined with great accuracy (Figure 2a).

The calculated concentration corresponded well to the adjusted cell count of 5*10^7 ^cells/ml for the mixed samples containing 25%, 50% and 75% of viable cells. Samples containing 0% and 100% viable cells showed a significant difference to the expected cell count of 5*10^7 ^cells/ml and were in a range between 5.57 * 10^7 ^- 6.65 * 10^7 ^cells/ml (Figure 2b).

### Plating

**Figure 3:**
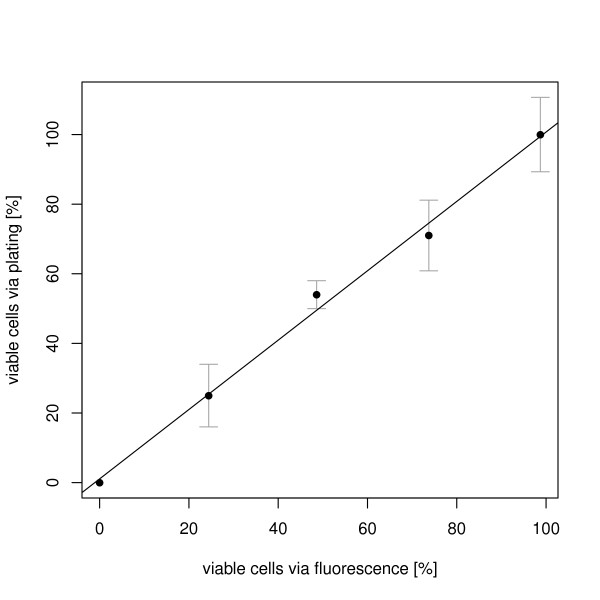
**Correlation between the number of viable cells as determined with plating and the cell-viability fluorescent assay**. The colony counts via plating and the number of fluorescent cells were determined in triplicates. The linear regression line between the two methods has a slope of 0.9865, intercept of 0.4618, and correlation coefficient of 0.9956.

Plating is the standard method to determine the number of viable cells in a culture. The different samples showed a decrease in the number of formed colonies with a decreasing number of viable cells in the mixture. A linear regression of the normalized count of colony forming cells on plates and the number of viable cells, as determined by fluorescence showed a correlation coefficient of 0.995 (Figure 3).

### Chlorophyll

**Figure 4:**
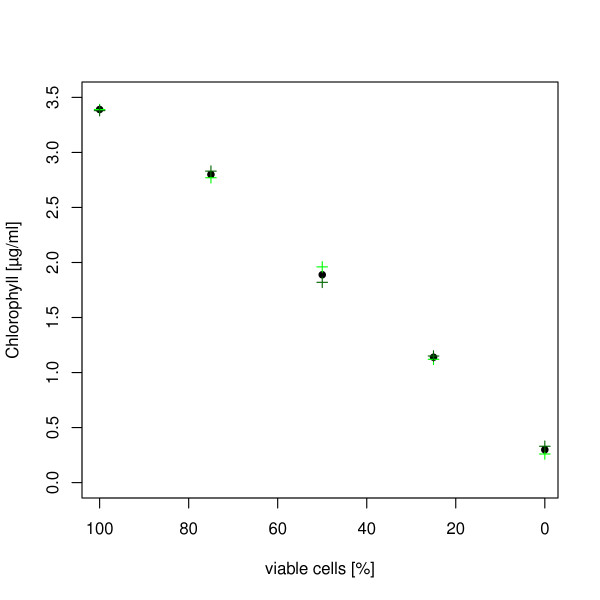
**Chlorophyll concentration for the different samples with an decreasing number of viable cells**. The Chlorophyll concentration was determined for every sample in duplicates. The graph shows the values of the two single measurements (green cross and dark green cross) as well as the mean concentration (black dot) plotted over the corresponding percentage of viable cells in the sample.

The chlorophyll concentration is often used as a measure for the viability of cyanobacteria cultures, and showed the expected decrease for the samples with decreasing number of viable cells (Figure 4).

### Absorption spectra

**Figure 5:**
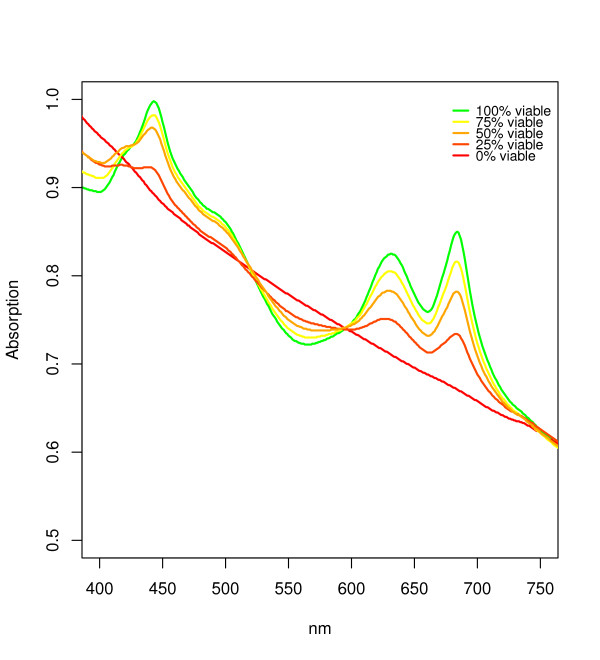
**Absorption spectra for the different samples with an decreasing number of viable cells**. The absorption spectra were recorded for the different samples in a range of 300 - 800 nm. All samples were adjusted to an optical density of 0.65. Plot limits were set to 400 and 750 nm since no additional information could be retrieved outside of these limits.

The determination of the absorption spectra is a conventional method to obtain an overview of the quantity and distribution of the pigments in the culture. In the sample with 100% viable cells the typical peaks for phycocyanin at 630 nm, chlorophyll a at 450 and 670 nm, and the carotenoids in the range of ~ 470-520 nm can be clearly seen (Figure 5). With increasing number of non-viable cells a decrease in the height of all peaks is clearly visible. For the sample with only non-viable cells no peaks at all can be detected, indicating a loss of all pigments.

### OD_750_

**Figure 6:**
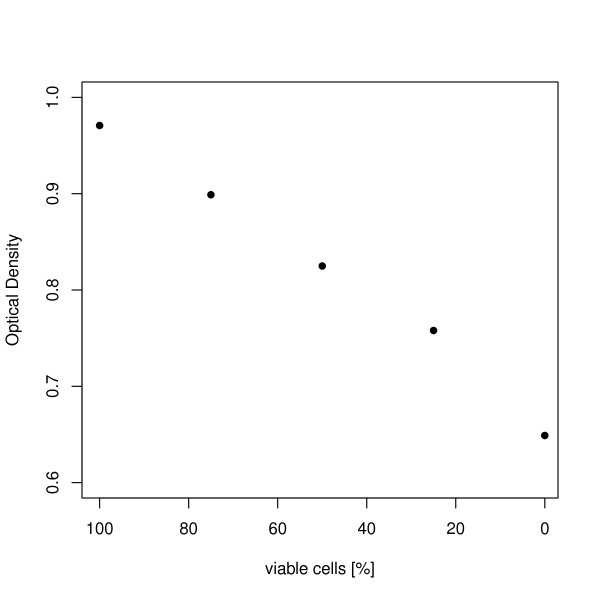
**Optical density at 750 nm for the different samples with an decreasing number of viable cells**. The measured optical density is plotted over the determined percentage of viable cells. Measurements were performed once for every sample.

The determination of the optical density is a very fast method and usually correlates well to the cell count of the culture. However, even though all samples were adjusted to a cell count of 5 * 10^7 ^cells per ml, the OD_750 _values showed a decrease from 0.971 to 0.649 with a decreasing number of viable cells (Figure 6).

### Applied vitality analysis on a cell by cell basis

Absorption spectra give a good overview over the content of pigments in the culture and are therefore used to determine the vitality of cultures. Nevertheless there can be different conditions of a culture that would result in the same absorption spectra. For example a culture with only stressed but viable cells could result in the same spectra as a culture that consists of a heterogeneous mix of unstressed viable and non-viable cells. Therefore an experiment was performed to test if a combination of absorption spectra and the new viability assay can be used to determine how many cells are contributing to the total pigmentation measured trough absorption. Thus allowing an estimation of the average pigmentation of the viable cells.

**Figure 7:**
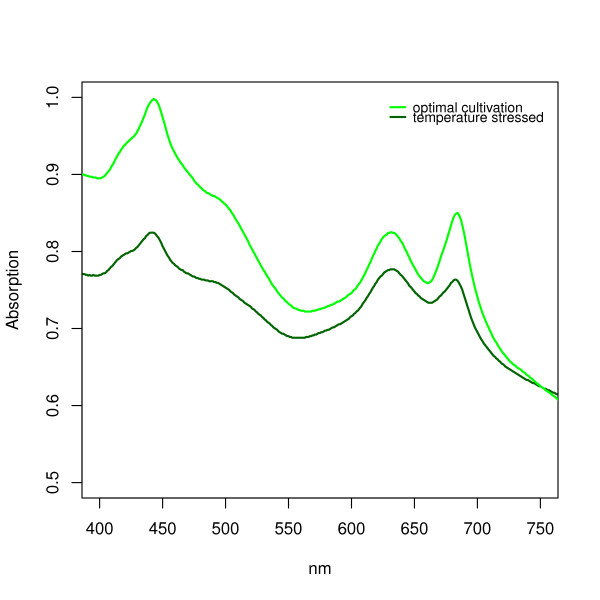
**Absorption Spectra for a temperature stressed and an optimally cultivated *Synechocystis *culture**. For both cultures a viability analysis was performed with the fluorescence assay and showed a difference of 9% in the number of viable cells (98% for the optimally cultivated culture and 89% for the temperature stressed culture).

To analyze this, the number of viable cells and an absorption spectrum were determined for a temperature adapted but stressed *Synechocystis *culture (Tillich, unpublished data; cultivation was in a bioreactor with day (12h): 44°C, 330 μE/m^2^*s, ph regulated CO (10%) (ph setpoint = 7.3); night (12h): 26°C; 10 ml/min bubbled air). The analyzed number of viable cells for the temperature stressed culture was 89% (only a difference of 9% to the 98% of the viable sample, see Figure 2). However, a comparison of the absorption spectra of both cultures showed a high difference in the total pigment content as determined by peak heights (Figure 7).

## Discussion

In this paper a new cell-viability fluorescence assay, which uses chlorophyll autofluorescence of viable cells and an unspecific green autofluorescence of non-viable cells, is presented for *Synechocystis*. It is a very simple and fast method without sample preparation or long growing phase for an immediate and direct analysis at the cellular level. Compared to staining methods it allows a more accurate analysis since contaminants (e.g. heterotrophic bacteria) in different cultures (data not shown) showed no fluorescent signal at the used excitation wavelength and are therefore not included in the analysis.

To validate the new method, results were compared to plating, chlorophyll concentration and absorption spectra, which are common methods for the determination of culture or cell viability. The strong correlation (R^2 ^= 0.995) between the percentage of viable cells as determined by the newly established autofluorescence method, and the number determined by plating confirm the results obtained with our method and show that an accurate viability analysis is possible.

The absorption spectra and chlorophyll concentration also matched the results of the viability analysis via fluorescence. The spectra for the culture, consisting only of non-viable cells, clearly shows that no photo pigments are present; therefore the green autofluorescence must be caused by other molecules. Additionally the sample consisting only of viable cells showed the highest peaks in the absorption spectra and concentration for chlorophyll.

These results seem to indicate that both, absorption spectra or chlorophyll concentration, can also be used for a quantification of viable cells. The measured values however are an average of all cells in the culture; therefore there is no distinction between part of the population dying or each cell reducing its pigment content while remaining viable. During cultivation there can be a number of different factors which lead to chlorophyll decrease within each cell [4]. Possible reasons include environmental factors such as high light stress or dying of a part of the population because of aging. Therefore absorption spectra and chlorophyll concentration are not suited for the analysis of the number of viable cells. This can only be achieved by a cell based approach like the presented fluorescence assay.

As mentioned before, the absorption spectra and the chlorophyll concentration give a good quantitative overview over the total amount of pigments in the culture. A combination with the newly developed binary method seems sensible. A comparison between the absorption spectra of a temperature stressed and an unstressed culture shows a much higher pigmentation for the unstressed culture, although the new viability assay shows a difference of the number of viable cells of only 9%. This indicates that the second culture has much lower pigment content per viable cell and these viable cells are therefore less vital (In this work the vitality of a culture/cell is defined as pigment content per culture/cell, since cyanobacteria show bleaching effects when they are grown under stressful culture conditions.). These results show that a combination of the new developed method and absorption spectra allows a more accurate quantification of the level of vitality of the culture. The combination of both methods makes it possible to determine the average pigmentation of the viable cells, since one can estimate the number of cells which are contributing to the overall signal measured photometrically.

Additionally cell counting results were compared to OD_750 _values of the different samples. The optical density is often used to determine the cell concentration of the culture. Our results however showed that although all samples had the same cell concentration, the OD_750 _values decreased with a decreasing viability of the culture. For the culture containing only non-viable cells the total cell concentration was underestimated by about 30% when using only OD_750_. This is probably caused by the loss of pigmentation, which results in changed absorption and light scattering characteristics of the culture. These findings indicate that a direct determination of the concentration via cell counting should be used when exact values are needed, since the relation between optical density and concentration is limited by the pigmentation of the cells.

The analysis of the cell concentrations with the presented automated cell counting showed significant differences to the expected cell count for the samples containing only viable and non-viable cells. These discrepancies are probably due to errors in the initial cell counting used for the dilution to a cell count of 5 * 10^7 ^cells, as this counting was made by hand to allow validation of the automated method. The samples consisting of mixtures of the viable and non-viable samples, showed concentrations that corresponded well to the expected cell count.

## Conclusions

In conclusion the newly developed method enables a quick and accurate quantification of viable and non-viable cells for *Synechocystis*. The method requires no staining and has virtually no costs in addition to the needed equipment. Results are obtained immediately, and match those obtained by (time consuming) plating. Beside the quantification of viable and non-viable cells, results indicate that one also can estimate the vitality of cells (e.g. via the course of a batch cultivation or before and after stressing conditions) by combining this method with absorption spectra or chlorophyll concentration measurements.

An adaptation of the auto fluorescence assay to a flow cytometer should also be possible. Beyond those of the possibility for an automated viability analysis of chlorophyll containing organisms without any staining, this would also allow the sorting in viable and non-viable cells (with a FACS). This could for example help to fasten the process of culture optimization.

## Methods

### Strain and culture conditions

*Synechocystis *sp. PCC 6803 was grown in BG11 medium (17,65 mM NaNO_3_, 0,18 mM K_2_HPO_4 _* 3H_2_O, 0,03 mM Citric acid, 0,003 mM EDTA (disodium magnesium), 0,19 mM Na_2_CO_3_, 0,03 mM Ferric ammonium citrate and trace metals) in a T75 tissue culture flask. Cultivation was in a Minitron shaker (INFORS HT) with photosynthesis lights at 35°C, 55 rpm and a continuous illumination of 20 μE/m^2^. The culture was kept in log growth phase by dilution.

### Preparation of viable and non-viable cells

A viable *Synechocystis *culture was split in two samples of 20 ml and OD_750 _= 2. To obtain non-viable cells, one sample was incubated at 60°C on a heated magnetic stirrer. The incubation was performed for 5 days in a closed Schott flask. The second sample was cultivated as previously described. The viable and non-viable culture was then counted in a Helber counting chamber to adjust the cell concentration to 5*10^7^cells per ml. Additionally the cultures were mixed in ratios of 1:0; 4:1; 2:1; 1:4 and 0:1.

### Semiautomated fluorescence microscopy for viability analysis

Cells were analyzed using a Helber counting chamber. To reduce cell movement within the chamber, the culture was mixed 1:2 with glycerol (99.5%, p.a.). Pretests with glycerol showed no influence to the cells and the fluorescence signal (data not shown). For image acquisition the automated Keyence BZ 9000 microscope and a chlorophyll fluorescence filter set (excitation: 435/40 nm; beam splitter: 510 nm; emission: 515 nm long-pass) were used. To enable a robust automated cell analysis, images were taken outside of the counting grid. Using the automated scanning module of the Keyence BZ II Viewer software, pictures of viable and non-viable fluorescence and phase contrast were taken for a volume of 0.084 μl. Four predefined corners of a square were focused by hand; using the "AutoRangePhoto" function of the software the focal points for all positions within the square were then extrapolated and pictures taken automatically.

For the automated viability analysis and cell counting, a plugin for ImageJ was written which is available in Additional file 1. To segment all cells from the background the fluorescent images were converted in a RGB-image stack. Afterwards automated thresholding with the "MaxEntropy" method [11] was used separately for both the red and green channel. Both images were combined and the cells were registered using the "Particle Analyzer" method [12]. Registered particles smaller then 35 μm^2 ^were excluded from the analysis to eliminate the influence of artefacts, which occurred due to image noise during thresholding of the image backgrounds (especially in the green channel). To differentiate between red and green cells the mean green-intensity of the cell area was thresholded at an intensity value of 50. This value was estimated by empiric testing. After all pictures were analyzed, the percentage of red and green cells, as well as the concentration per ml was calculated. These experiments were performed three times for every sample.

### OD_750 _measurement and absorption spectroscopy

The optical density of the samples was measured at 750 nm using the UV-Spectrophotometer UV-1800 (Shimadzu). For the analysis of the absorption, the cultures were adjusted to an optical density of 0.65, and spectra were recorded in the range of 300 - 800 nm.

### Chlorophyll measurement

According to [1] 2 ml of the culture were centrifuged at 15.000 g for 10 min in a 2 ml reaction tube. 1900 μl of the supernatant were removed and the cells were resuspended using an ultrasonic bath. Afterwards 900 μl of 100% methanol were added and incubated in the dark at +4°C for 1 hour. Samples were then centrifuged at 15.000g for 10 minutes and absorption was measured at 650 nm. Chlorophyll concentration in μg/ml was calculated by the following formula:

$$\text{Chlorophyll}\;\left(\mu \text{g}∕\text{ml}\right)=\left(\text{Ab}{\text{s}}_{650}\;*\;13.9\right)∕2\text{ml}$$

The chlorophyll measurement was performed two times for every sample.

### Cell plating

Viable cells per ml were determined by spreading 5*10^4 ^cells of the samples on agar plates (87 mm inner diameter; 60.1 cm^2 ^growth area) containing BG-11 and 1% agar. The plates were incubated for one week at room temperature and 10 μE/m^2^s. Colonies were counted using the autofluorescence of phycocyanin. Fluorescence pictures of the plates were taken with the Keyence BZ9000 and a phycocyanin fluorescence filter set (excitation: 600/37 nm; beam splitter: 625 nm; emission: 655/40 nm). Using the automated scanning module of the Keyence Software, pictures of an area of 10.5 cm^2 ^were taken. Automated counting was done by another self written ImageJ plugin which uses histogram thresholding for segmentation and the integrated "Analyze Particles" function. To calculate the viability of the cultures the colony count of all samples was normalized by the colony count of the control (only viable cells plated). The plating was performed in triplicates for every sample.

## Authors' contributions

KS conceived the method, established the automation for the microscopy and the image analysis via ImageJ and wrote the manuscript. DAL and UMT designed the experimental work. DAL and KS performed the experiments. UMT contributed to the writing of the manuscript and performed culturing of the temperature stressed culture. MF supervised the research, provided laboratory facilities and contributed to the writing of the manuscript. All authors read and approved the final manuscript.

## Supplementary Material

Additional file 1 **ImageJ plugin and sample pictures**. The plugin can be used for an automated analysis of microscopic images of the new cell-viability fluorescence assay. ImageJ is required and can be downloaded from http://rsbweb.nih.gov/ij/download.html. For an installation of the plugin extract the .jar file into the plugin folder and restart ImageJ. The plugin can be found under Plugins > LivingDead. Example images for the plugin can be found in the folder ExampleImages. Click here for file

## References

[B1] Tandeau de Marsac N Occurrence and nature of chromatic adaptation in cyanobacteria J Bacteriol 1977 130 82 91 856789 10.1128/jb.130.1.82-91.1977 PMC235176

[B2] Sato M Murata Y Mizusawa M A Simple and Rapid Dual-fuorescence Viability Assay for Microalgea Microbiol Cult Coll 2004 20 2 53 59

[B3] Lee TJ Nakano K Matsumura M A new method for the rapid evaluation of gas vacuoles regeneration and viability of cyanobacteria by flow cytometry Biotechnology Letters 2000 22 1833 1838 10.1023/A:1005653124437

[B4] Beck E Scheibe R Senescence and ageing in plants and cyanobacteria Physiologia Plantarum 2003 119 1 4 10.1034/j.1399-3054.2003.00140.x

[B5] Hörtensteiner S Chlorophyll degradation during senescence Annual review of plant biology 2006 57 55 77 10.1146/annurev.arplant.57.032905.105212 16669755

[B6] Hense B Gais P Jutting U Scherb H Rodenacker K Use of fluorescence information for automated phytoplankton investigation by image analysis Journal of Plankton Research 2008 30 5 587 606 10.1093/plankt/fbn024

[B7] Tang YZ Dobbs FC Green autofluorescence in dinoflagellates, diatoms, and other microalgae and its implications for vital staining and morphological study Appl Environ Microbiol 2007 73 2306 2313 10.1128/AEM.01741-06 17277199 PMC1855647

[B8] Abramoff M Magalhaes P Ram S Image Processing with ImageJ Biophotonics International 2004 11 7 36 42

[B9] Rasband W ImageJ U. S. National Institutes of Health, Bethesda, Maryland, USA 1997 2011 http://imagej.nih.gov/ij/

[B10] Kaneko T Sato S Kotani H Tanaka A Asamizu E Nakamura Y Miyajima N Hirosawa M Sugiura M Sasamoto S Kimura T Hosouchi T Matsuno A Muraki A Nakazaki N Naruo K Okumura S Shimpo S Takeuchi C Wada T Watanabe A Yamada M Yasuda M Tabata S Sequence analysis of the genome of the unicellular cyanobacterium Synechocystis sp. strain PCC6803. II. Sequence determination of the entire genome and assignment of potential protein-coding regions DNA research 1996 3 3 185 209 10.1093/dnares/3.3.185 8905238

[B11] Kapur JN Sahoo PK Wong ACK A New Method for Gray-Level Picture Thresholding Using the Entropy of the Histogram Graphical Models and Image Processing 1985 29 3 273 285

[B12] Igathinathane C Pordesimo LO Columbus EP Batchelor WD Methuku SR Shape identification and particles size distribution from basic shape parameters using ImageJ Computers and Electronics in Agriculture 2008 63 2 168 182 10.1016/j.compag.2008.02.007

