# Supplementary figures and images for: A simple viability analysis for unicellular cyanobacteria using a new autofluorescence assay, automated microscopy, and ImageJ
Source: BMC Biotechnol. 2011 Nov 30;11:118. doi: 10.1186/1472-6750-11-118 (PMC3247844; doi:10.1186/1472-6750-11-118)

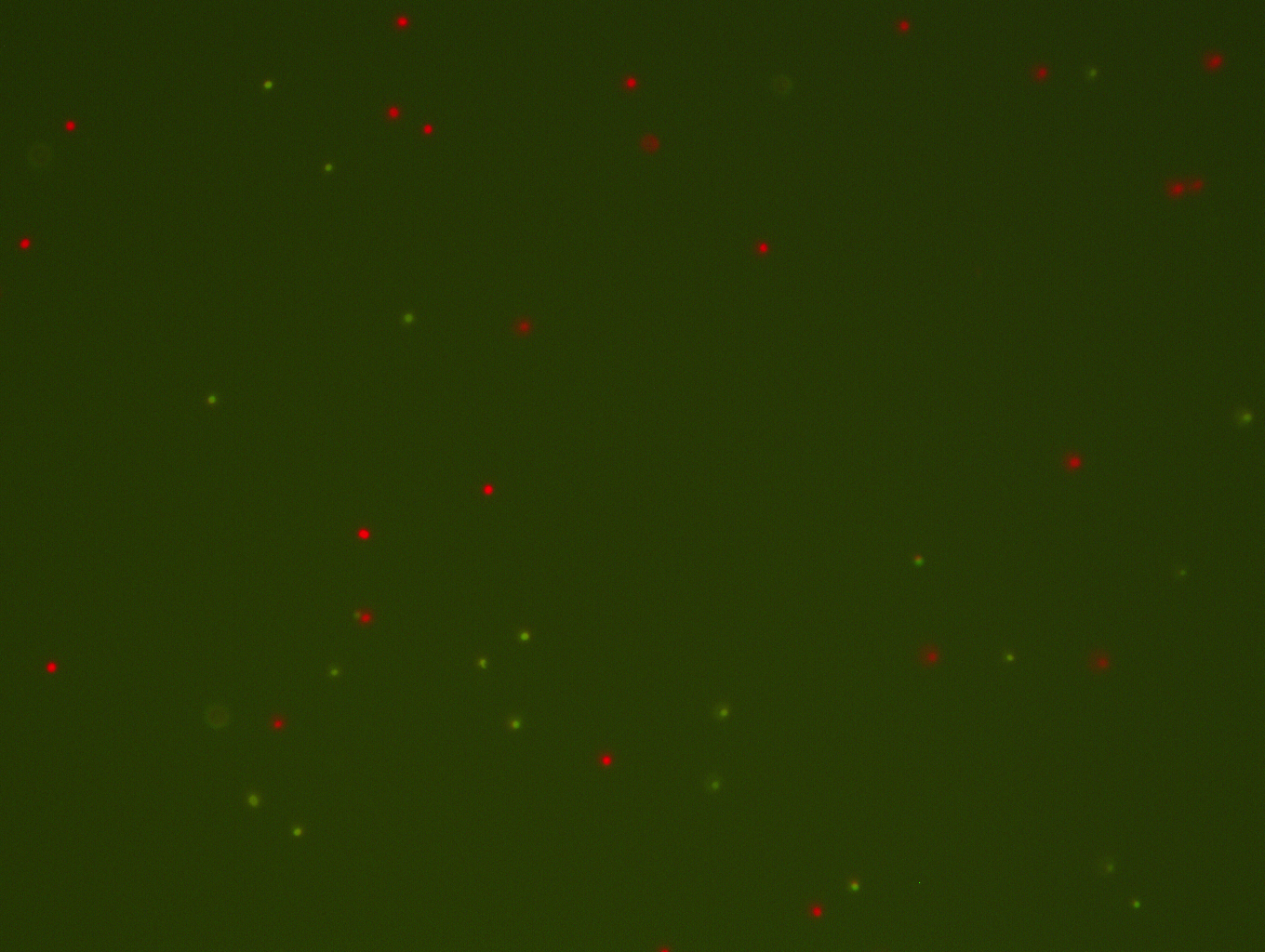

Supplement: Additional file 1 — ImageJ plugin and sample pictures. The plugin can be used for an automated analysis of microscopic images of the new cell-viability fluorescence assay. ImageJ is required and can be downloaded from http://rsbweb.nih.gov/ij/download.html. For an installation of the plugin extract the .jar file into the plugin folder and restart ImageJ. The plugin can be found under Plugins > LivingDead. Example images for the plugin can be found in the folder ExampleImages. [file 1472-6750-11-118-S1.ZIP › LivingDead_Plugin/ExampleImages/3.BMP]

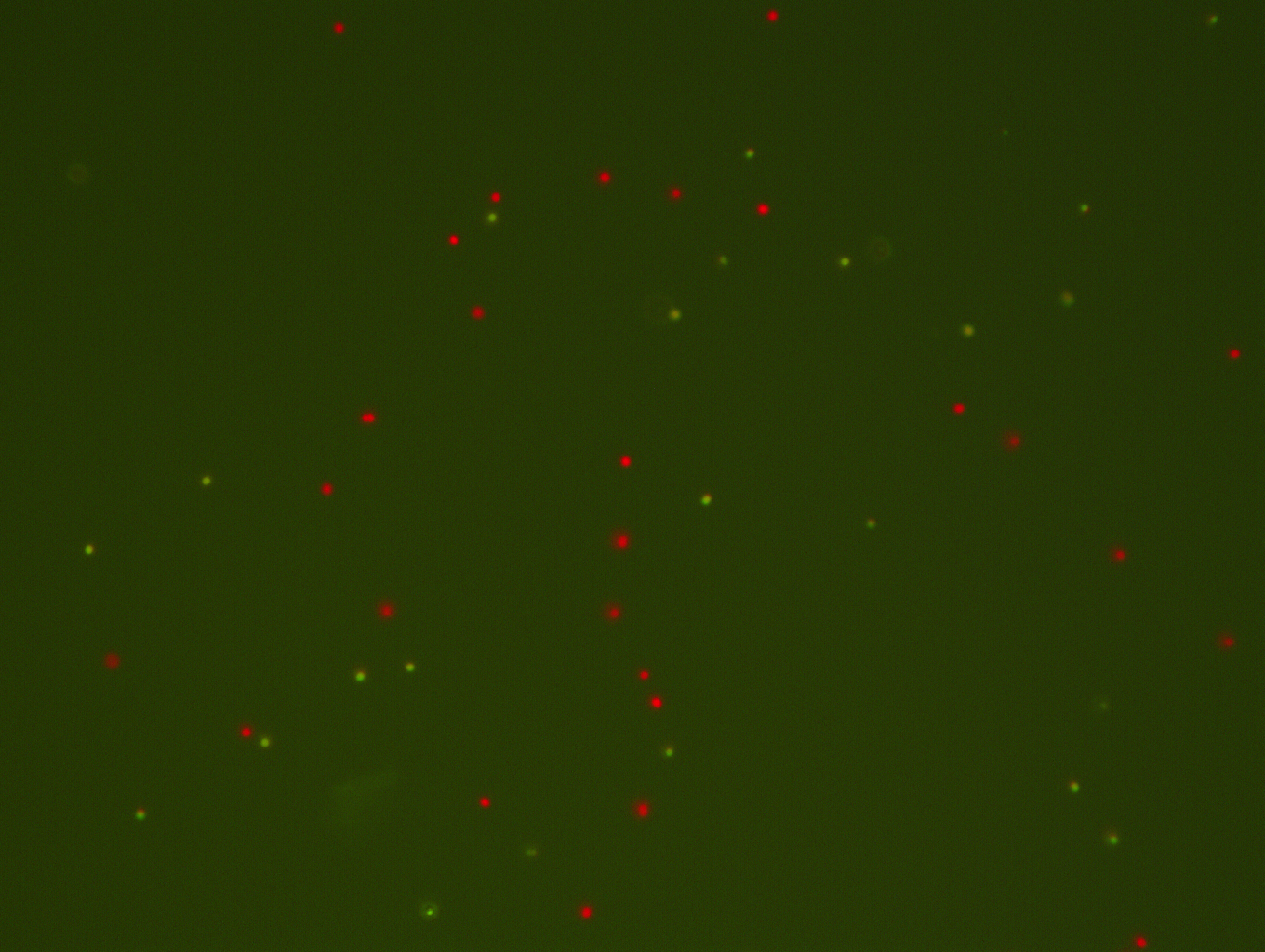

Supplement: Additional file 1 — ImageJ plugin and sample pictures. The plugin can be used for an automated analysis of microscopic images of the new cell-viability fluorescence assay. ImageJ is required and can be downloaded from http://rsbweb.nih.gov/ij/download.html. For an installation of the plugin extract the .jar file into the plugin folder and restart ImageJ. The plugin can be found under Plugins > LivingDead. Example images for the plugin can be found in the folder ExampleImages. [file 1472-6750-11-118-S1.ZIP › LivingDead_Plugin/ExampleImages/2.BMP]

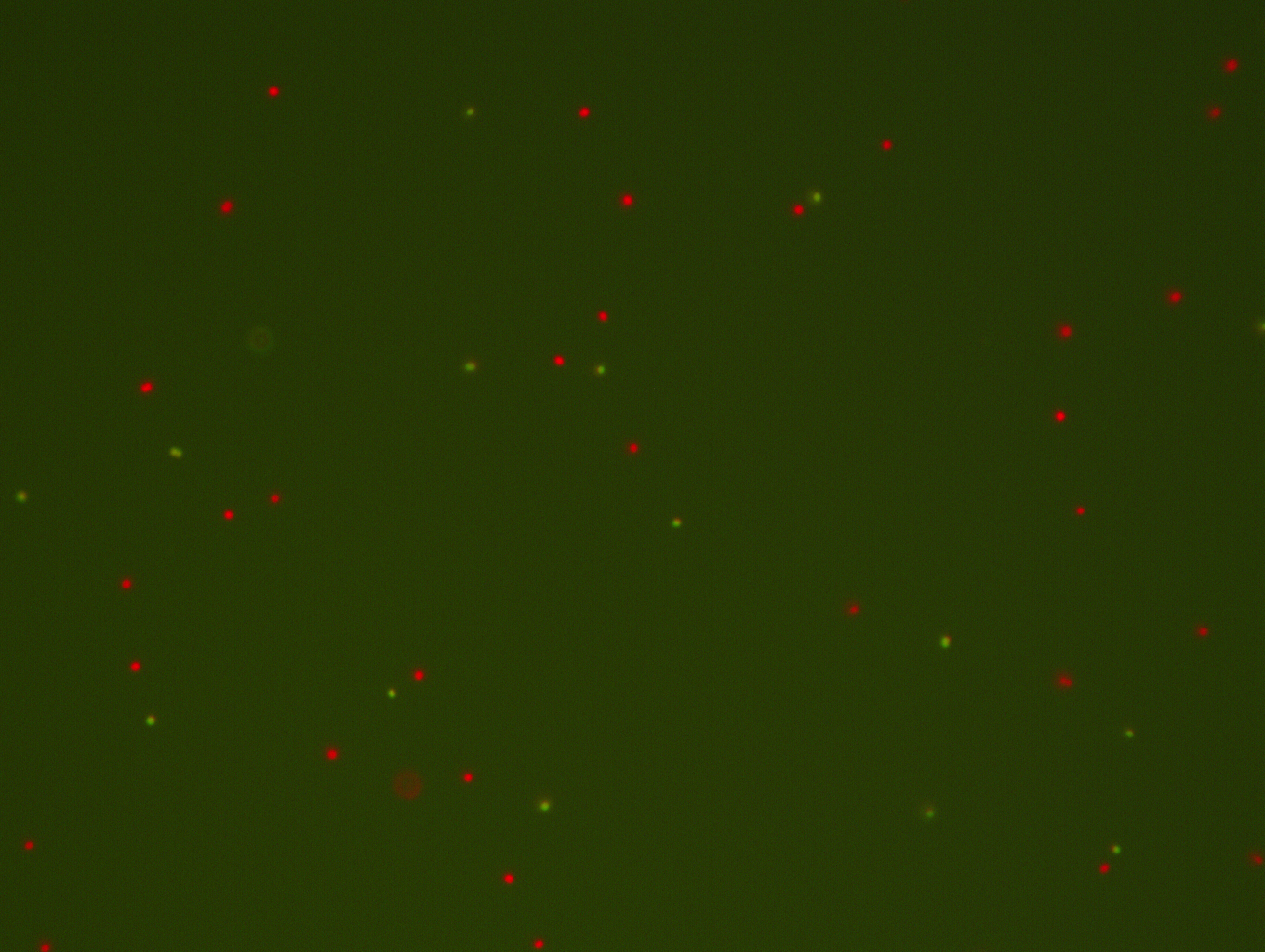

Supplement: Additional file 1 — ImageJ plugin and sample pictures. The plugin can be used for an automated analysis of microscopic images of the new cell-viability fluorescence assay. ImageJ is required and can be downloaded from http://rsbweb.nih.gov/ij/download.html. For an installation of the plugin extract the .jar file into the plugin folder and restart ImageJ. The plugin can be found under Plugins > LivingDead. Example images for the plugin can be found in the folder ExampleImages. [file 1472-6750-11-118-S1.ZIP › LivingDead_Plugin/ExampleImages/1.BMP]
